# Immune Mechanism, Gene Module, and Molecular Subtype Identification of Astragalus Membranaceus in the Treatment of Dilated Cardiomyopathy: An Integrated Bioinformatics Study

**DOI:** 10.1155/2021/2252832

**Published:** 2021-09-14

**Authors:** Xiao-yang Chen, Hong-fei Han, Zhen-yan He, Xue-gong Xu

**Affiliations:** ^1^Graduate School of Henan University of Chinese Medicine, Zhengzhou 450046, China; ^2^Neurosurgery Department, Henan Cancer Hospital, Zhengzhou 450008, China; ^3^Cardiovascular Department, Zhengzhou Hospital of Traditional Chinese Medicine, Zhengzhou 450007, China; ^4^Laboratory of Zhengzhou Hospital of Traditional Chinese Medicine, Zhengzhou 450007, China

## Abstract

Astragalus membranaceus has complex components as a natural drug and has multilevel, multitarget, and multichannel effects on dilated cardiomyopathy (DCM). However, the immune mechanism, gene module, and molecular subtype of astragalus membranaceus in the treatment of DCM are still not revealed. Microarray information of GSE84796 was downloaded from the GEO database, including RNA sequencing data of seven normal cardiac tissues and ten DCM cardiac tissues. A total of 4029 DCM differentially expressed genes were obtained, including 1855 upregulated genes and 2174 downregulated genes. GO/KEGG/GSEA analysis suggested that the activation of T cells and B cells was the primary cause of DCM. WGCNA was used to obtain blue module genes. The blue module genes are primarily ADCY7, BANK1, CD1E, CD19, CD38, CD300LF, CLEC4E, FLT3, GPR18, HCAR3, IRF4, LAMP3, MRC1, SYK, and TLR8, which successfully divided DCM into three molecular subtypes. Based on the CIBERSORT algorithm, the immune infiltration profile of DCM was analyzed. Many immune cell subtypes, including the abovementioned immune cells, showed different levels of increased infiltration in the myocardial tissue of DCM. However, this infiltration pattern was not obviously correlated with clinical characteristics, such as age, EF, and sex. Based on network pharmacology and ClueGO, 20 active components of Astragalus membranaceus and 40 components of DMCTGS were obtained from TCMSP. Through analysis of the immune regulatory network, we found that Astragalus membranaceus effectively regulates the activation of immune cells, such as B cells and T cells, cytokine secretion, and other processes and can intervene in DCM at multiple components, targets, and levels. The above mechanisms were verified by molecular docking results, which confirmed that AKT1, VEGFA, MMP9, and RELA are promising potential targets of DCM.

## 1. Introduction

Dilated cardiomyopathy (DCM) is a heterogeneous type of cardiomyopathy characterized by ventricular enlargement and reduced myocardial systolic function, excluding hypertension, valvular heart disease, congenital heart disease, and ischaemic heart disease [1]. A stratified cluster sampling survey of 8080 individuals in the general population in 9 regions of China in 2002 revealed a DCM prevalence of 19/100,000 [2]. According to a 2014 Chinese report, the case fatality rate of 767 DCM patients from follow-up for 52 months was 42.24%, conveying a heavy burden to society and family [3]. DCM can occur at any age, with a high incidence in those 20–50 years old and affecting more males than females (2.5 : 1), and its incidence has been on the rise in recent years [4]. The etiology of this disease is unclear, and genetics, infection, immunity, and other issues can cause this disease. Its pathophysiological mechanism is complex, and its clinical manifestations include cardiac enlargement, arrhythmia, thromboembolism, heart failure, and sudden death [5–7]. Twenty to 35% of DCM patients exhibit a typical genetic basis. The related pathogenic genes primarily encode myosin, ion channel proteins, nuclear layer proteins, structural proteins, etc. Nevertheless, the genetic basis of a large portion of DCM patients remains unclear [8, 9]. Differences in disease phenotypes caused by mutations in the same gene increase the complexity of this disease [10, 11].

At present, DCM is primarily classified according to clinical symptoms, focusing on evaluating the cardiac function [12]. DCM is classified as hereditary, mixed, and acquired by the American Heart Association (AHA). In contrast, cardiomyopathy is classified as familial (hereditary) and nonfamilial (nonhereditary) by the European Society of Cardiology (ESC) according to the causes and stages of the disease [13, 14]. Studies have shown that DCM has different pathological mechanisms during different stages and distinct aetiologies, exhibiting evident heterogeneity and completely different courses and prognoses [15–17]. However, the above classification or classification methods ignored molecular subtypes, which leads to inaccurate clinical treatment. Different clinical molecular subtypes often need varied intervention methods [18, 19]. Studies have shown that DCM is closely related to immunity, including the T-cell-mediated inflammatory response, as well as myocardial infiltration of macrophages and neutrophils, leading to the progression of cardiac fibrosis [20]. Although currently developed single-cell sequencing and traditional immunohistochemical staining methods have made it possible to elucidate the immune infiltration pattern, it is still difficult to estimate subpopulations with low cell abundance; therefore, a complete and specific pattern of immune infiltration ha not yet been reported [21]. Current immunotherapies, such as immunoadsorption and immunosuppression, have achieved some results, but their accuracy and clinical safety remain questionable [22].

Traditional Chinese medicine (TCM) is a matured systematic medicine with a history of more than 2000 years [23]. Dilated cardiomyopathy has a long history in TCM, traced back to *Huangdi's Inner Canon-Su Wen* in the pre-Qin period [24]. Astragalus membranaceus, a commonly used Chinese medicine, is considered to improve dyspnoea, fatigue, and even lower extremity edema and other symptoms of DCM [25, 26]. Modern studies have shown that many components of Astragalus membranaceus, such as Astragalus polysaccharide and Astragalus IV, play roles in immune regulation and cardiovascular protection [27]. Astragalus membranaceus has complex components as a natural drug and has multilevel, multitarget, and multichannel effects on dilated cardiomyopathy. Although there are relevant network pharmacology studies on the possible mechanism of action, the data used in them are broad, which may lead to prediction results that are inconsistent with the actual situation. Furthermore, the immune-related mechanisms have not been systematically analyzed [28]. Based on these findings, this study used integrated bioinformatics technology to identify molecular subtypes of DCM, the immune invasion mode, and the gene module and to explore the networked model of immune regulation of Astragalus membranaceus in DCM.

## 2. Materials and Methods

### 2.1. Data Processing

Seventeen patients in GSE84796 were downloaded from the Gene Expression Omnibus (GEO). We used the “SVA” package in R for batch correction [29]. Data on the clinical traits of microarray samples were derived from previously published articles [30]. The components and target genes of Chinese medicine were acquired from the TCMSP database [31].

### 2.2. GO/KEGG Analysis and Gene Set Enrichment Analyzes (GSEA)

Differentially expressed genes (DEGs) were calculated and labeled using the *Limma* package. Subsequently, DEGs were analyzed by gene ontology (GO) and Kyoto Encyclopedia of Genes and Genomes (KEGG) pathway analyzes [32–34]. GO analysis consists of three parts: molecular function (MF), biological process (BP), and cell component (CC). *P* < 0.05 was defined as statistically significant. GSEA was performed using GSEA software (version 4.0.3) [35].

### 2.3. Weighted Gene Coexpression Network Analysis (WGCNA) and Molecular Subtype Recognition

In order to identify modules highly correlated with DCM, WGCNA was performed using the WGCNA R package and on all genes [36]. The Pearson correlation coefficient was used to establish an unsupervised coexpression relationship based on the connection strength adjacency matrix for gene pairs. This matrix was increased based on the scale-free topology criterion [37]. Then, the topologically overlapping matrix was used to analyze the adjacency matrix of clustered GC patient gene expression data. Finally, the dynamic tree cut algorithm was applied to the dendrogram for module identification using the minisize module gene numbers set as 50 and a cut height of 0.9. In the module-trait analysis, GS values > 0.3 and MM values > 0.55 were defined as thresholds. The modules related to clinical traits were subsequently identified and imported into the STRING database (https://string-db.org/) for PPI network analysis [38, 39]. After using core genes to identify molecular subtypes, parting was performed using the ConsensusClusterPlus package (http://www.bioconductor.org/).

### 2.4. Examining Immune Infiltration Using CIBERSORT Analysis

The CIBERSORT algorithm was used to analyze the normalized gene expression data obtained previously, and the proportions of 22 kinds of immune cells were determined [40]. These immune cells included naive B cells, memory B cells, plasma cells, CD8+ T cells, naive CD4+ T cells, resting memory CD4+ T cells, activated memory CD4+ T cells, follicular helper T cells, regulatory T cells (Tregs), gamma delta T cells, resting NK cells, activated NK cells, monocytes, M0 macrophages, M1 macrophages, M2 macrophages, resting dendritic cells, activated dendritic cells, resting mast cells, activated mast cells, eosinophils, and neutrophils. The samples were screened according to a *P* value <0.05, and the percentage of each type of immune cell in the samples was calculated. Principal component analysis (PCA) was performed to determine whether there was a difference in immune cell infiltration between the myocardial tissue of DCM patients and that of normal controls. The different immune infiltration levels of each immune cell type between the two groups were analyzed using the vioplot package in R version 3.6.0. The correlation among the immune cells was analyzed using the corrplot package. At the same time, Wilcoxon correlation analysis was performed between the pattern of immune infiltration and clinical traits, such as age, sex, and EF value.

### 2.5. The Pharmacological Mechanism of Astragalus in the Treatment of DCM

The effective component targets of Astragalus membranaceus intersected with DEGs, and the common drug and disease genes were imported into STRA to construct a PPI network. The species selected were *Homo sapiens*, and the interaction relationship between the target proteins was obtained. After the PPI file was exported, Cytoscape was used to optimize the graph, and the top four core genes were obtained according to the degree value for subsequent verification [41]. The GO/KEGG enrichment analysis of common drug-related genes was performed using the DAVID database [42]. The immune function of Cytoscape's ClueGo plug-in was used to analyze the immune mechanism of Astragalus interfering with DCM, and a specific network of immune regulation mechanisms was constructed [43]. Finally, Cytoscape was used to construct a network diagram of the active component-action target-signal pathway of Astragalus intervention in DCM.

### 2.6. Molecular Docking

Discovery Studio (DS, V2016) is a new generation of molecular simulation software that uses the dynamics method of the CDOCKER module to randomly search for small molecule conformations and then uses a simulated annealing method to optimize each conformation in the receptor active site region [44–46]. Therefore, in this study, DS was used to perform molecular docking experiments on the effective components of Astragalus membranaceus and the core target protein of disease drugs. The specific methods were as follows: (1) the hub genes were searched on the RCSB PDB website (https://www.rcsb.org/search), and PDB structural formulas were obtained [47]; (2) the ligands were introduced into DS software, and the function “prepare ligands” was used to prepare the ligands. At the same time, water, ligand groups, and irrelevant side chains were removed from receptors, and receptor-ligand interactions >define and edit binding site > from receptor cavities were selected to identify potential active sites in protein cavities; (3) the CDOCKER function was used to perform molecular docking, and according to -CDOCKER _ INTERACTION _ ENERGY (negative molecular docking binding energy), the molecular conformation with the best score in each docked molecule was obtained and ranked [48]. The results showed that the higher the binding energy of the docking conformation, the more stable the binding conformation, indicating a greater possibility of binding between the reaction receptor molecule and the ligand.

## 3. Results

### 3.1. Gene Chip Data Annotation and TCM Information Acquisition

**Figure 1:**
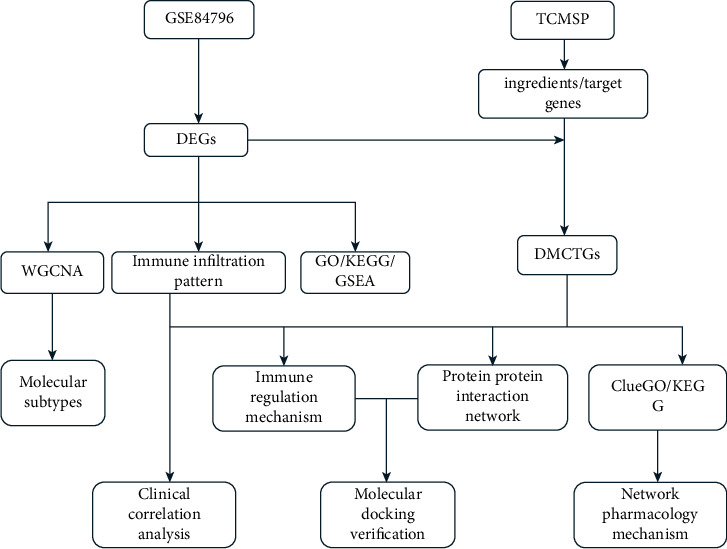
Research technology roadmap.

**Figure 2:**
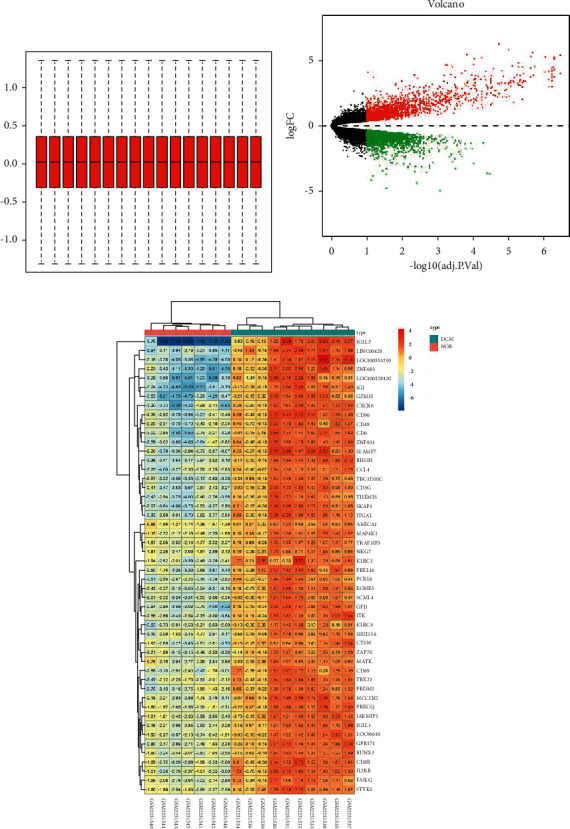
Correction and difference analysis of gene chip information.

The research design process for this study is shown in Figure 1. First, we downloaded the microarray information of GSE84796 from the GEO database, including RNA sequencing data of 7 normal cardiac tissues and 10 DCM cardiac tissues. Then, batch correction and difference analysis were performed using the so-and-so package in R language (Figures 2(a) and 2(b)). As a result, 4029 differential genes were obtained, including 1855 upregulated genes and 2174 downregulated genes (2C). Next, the composition information of Astragalus membranaceus was downloaded from the TCMSP database, and the active ingredients were screened according to the five principles of drug class (OB>30%, DL > 0.18), resulting in a total of 20 active ingredients obtained, as shown in Table 1 [49, 50]. Then, the target information of each component was obtained and transformed into the corresponding GeneSymbol using the UniProt website for a total of 190 DEGs.

### 3.2. GO/KEGG and GSEA Enrichment Analysis of DEGs

**Figure 3:**
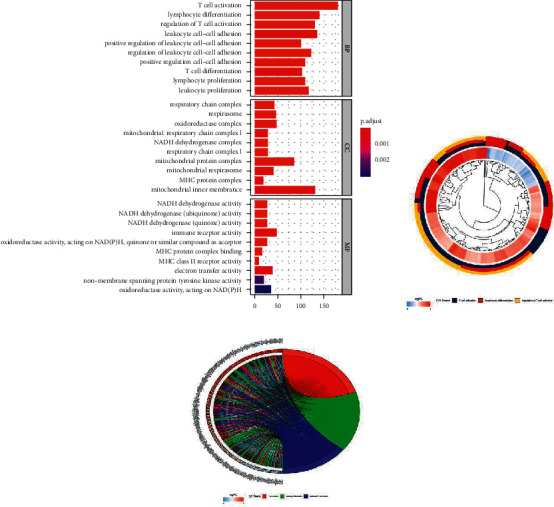
Go enrichment analysis of differentially expressed genes.

**Figure 4:**
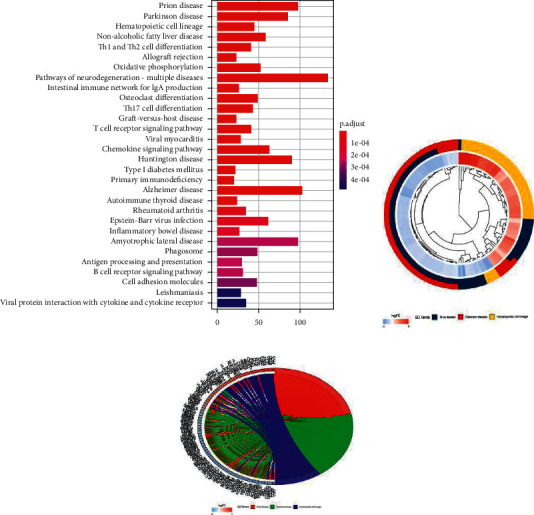
KEGG enrichment analysis of differentially expressed genes.

**Figure 5:**
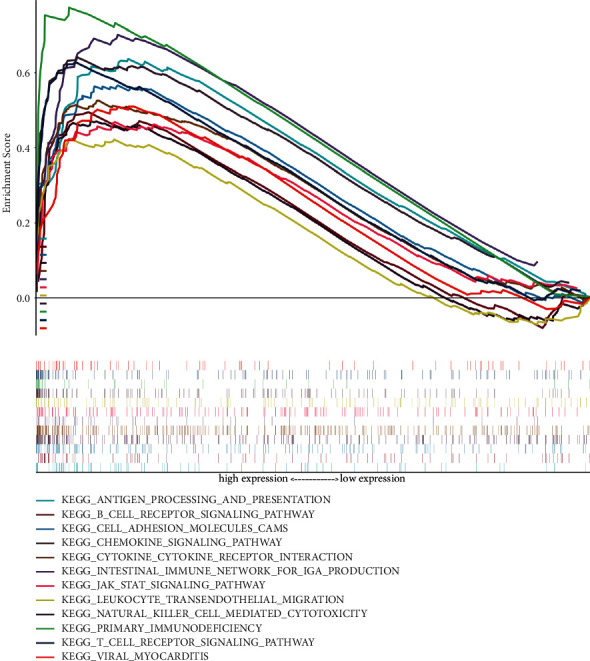
GSEA enrichment analysis of differentially expressed genes.

Online GO/KEGG enrichment analysis of DEGs was performed using the R language package, and ranking was performed according to the *P* value, as shown in Figures 3 and 4. GO enrichment includes BP, CC, and MF. It primarily revealed NADH dehydrogenase activity, NADH dehydrogenase (ubiquinone) activity, NADH dehydrogenase (quinone) activity, immune receptor activity, and other molecular functions, which are primarily involved in BP cellular components such as respiratory chain complexes, respirasomes, oxidoreductase complexes, and mitochondrial respiratory chain complex I, primarily participating in T cell activation, including lymphocyte differentiation and regulation of T cell activation. KEGG was primarily enriched in the T cell receptor signaling pathway, graft versus host disease, Th17 cell differentiation, osteoblast differentiation, the internal immune network for IgA production, pathways of neurodegeneration, multiple diseases, and other signaling pathways. GO/KEGG enrichment analysis fails to consider the amount of gene expression in differential gene analysis. In contrast, GSEA fully considers gene expression and integrates the existing information of gene location, nature, function, and biological significance to analyze the entire genome expression profile chip data. The results of GSEA enrichment analysis are shown in Figure 5, which are primarily enriched in antigen processing and presentation, cell adhesion molecules (CAMs), chemical signaling pathway, T cell receiver signaling pathway, B cell receiver signaling pathway, the internal immune network for IgA production, primary immunodeficiency, natural killer cell modified cytotoxicity, and other signaling pathways, and some additional immune-related signaling pathways.

### 3.3. WGCNA and Molecular Subtype Recognition

**Figure 6:**
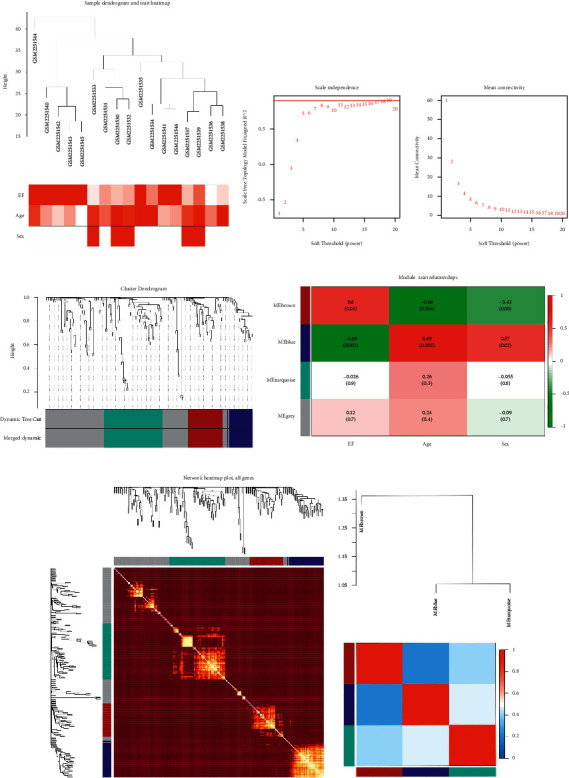
WGCNA analysis and gene module identification.

**Figure 7:**
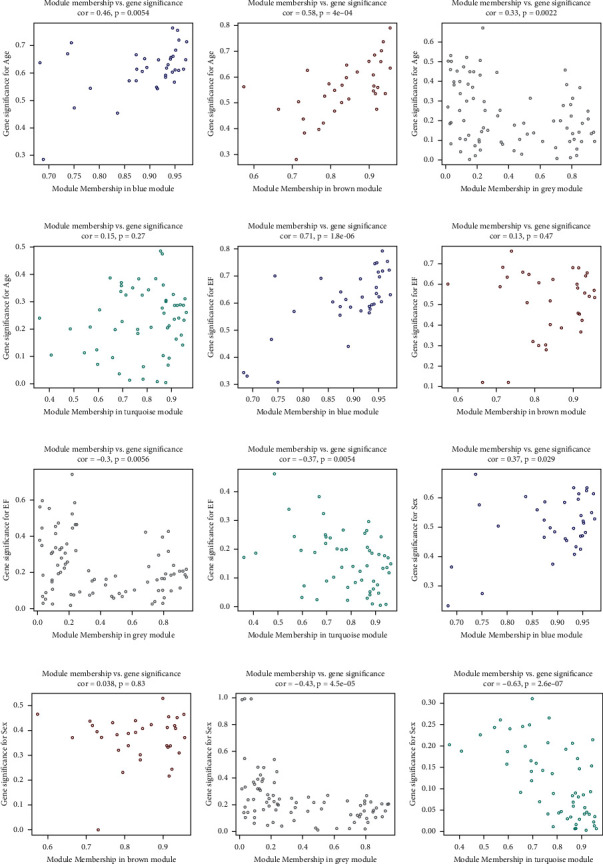
Correlation between modular genes and clinical traits.

A sample tree diagram and characteristic heat map were used to identify the basic features of the sample (Figure 6(a)). According to the scale-free fitting index and average connectivity analysis, WGCNA determined that the soft threshold *β* was 18. When the scale-free index reached 0.76, the conditions for constructing the scale-free network were satisfied (Figure 6(b)). Then, the Pearson matrix was transformed into an adjacency matrix and replaced with the corresponding topological overlap matrix to obtain four different modules (Figure 6(c)). Figure 6 shows the visualization of genes and colors within the module. The upper part is a cluster tree of genes, while the lower part displays modules in different colors, including blue, brown, green, and grey, which contain 35, 33, 55, and 84 genes, respectively. Genes that could not be entered into any of the coexpressed modules are shown in grey. According to the correlation analysis between the gene module and the clinic, the blue module was highly correlated with age (COR = 0.46, *P* = 0.0054), EF (COR = 0.74, *P* = 1.8*E* − 06), and sex (COR = 0.37, *P* = 0.029) (Figures 6(d) and 7). Moreover, there was no strong correlation between any gene modules (Figures 6(e) and 6(f)).

**Figure 8:**
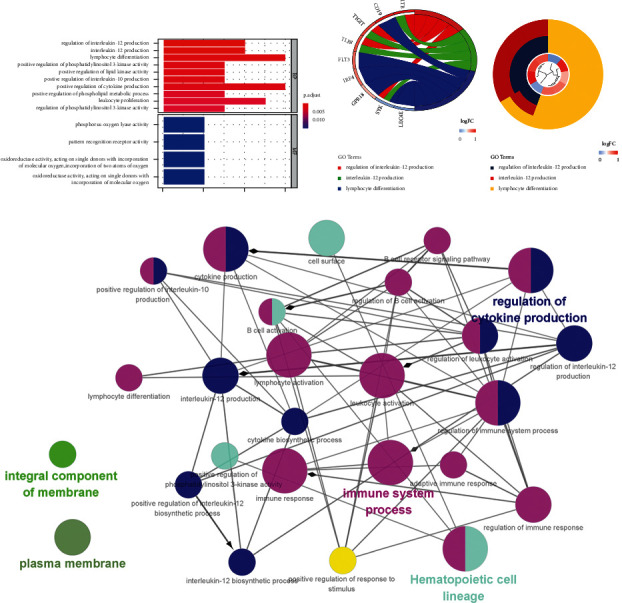
GO/KEGG enrichment analysis of blue module genes.

**Figure 9:**
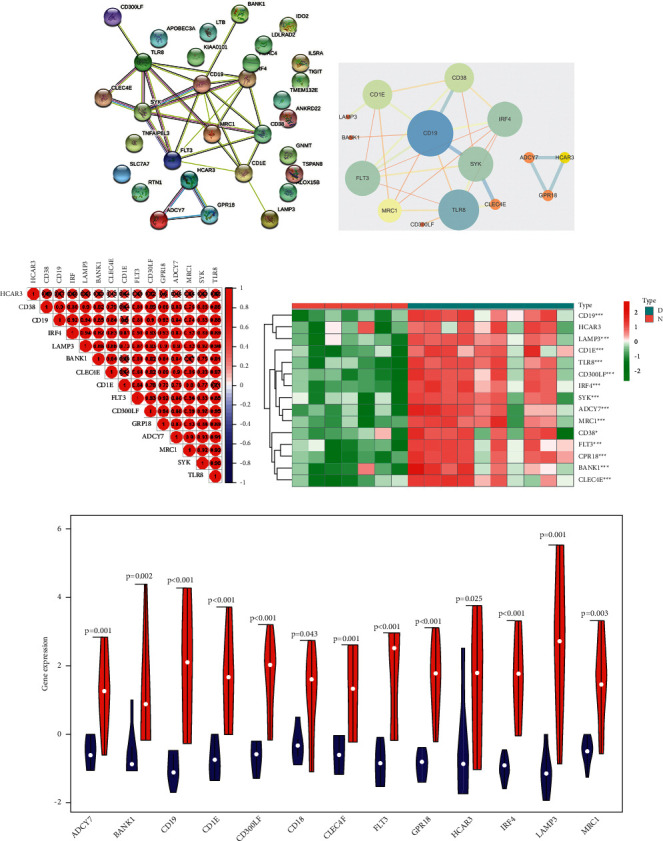
Hub genes from PPI network of the blue module.

**Figure 10:**
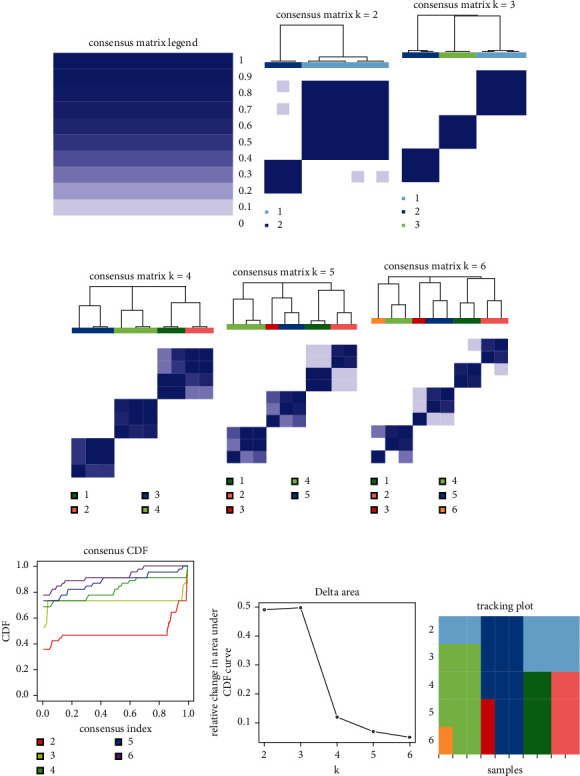
Molecular subtype identification by hub genes of the blue module.

The results of the enrichment analysis of the blue module genes were primarily related to the regulation of interleukin-12 production, differentiation, position regulation of phosphatidylinositol 3-kind activity, position regulation of lip type activity, position regulation of interleukin-10 production, and the phosphorus oxygen ASE activity, and MF was related to the Peter recognition receiver activity (Figures 8(a)–8(c)). KEGG was primarily enriched in hematopoietic cell lineage and other signaling pathways. The complex correlation between each GO/KEGG enrichment item indicates that the 15 genes of this cluster are related to each other in physiological interactions, and most of them are closely related to immunity, inflammation, etc. (Figure 8(d)). Fifteen hub genes were extracted from the blue module: adyy7, bank1, ADCY7, BANK1, CD1E, CD19, CD38, CD300LF, CLEC4E, FLT3, GPR18, HCAR3, IRF4, LAMP3, MRC1, SYK, and TLR8 (Figures 9(a) and 9(b)). These genes were ranked according to degree and showed good correlation and significant differences between patients and controls (Figures 9(c)–9(e)). Next, the 15 genes were used to classify the samples, and these 15 genes divided DCM into three subtypes with clear divisions, as shown in Figure 10.

### 3.4. Analysis of Immune Infiltration Pattern

**Figure 11:**
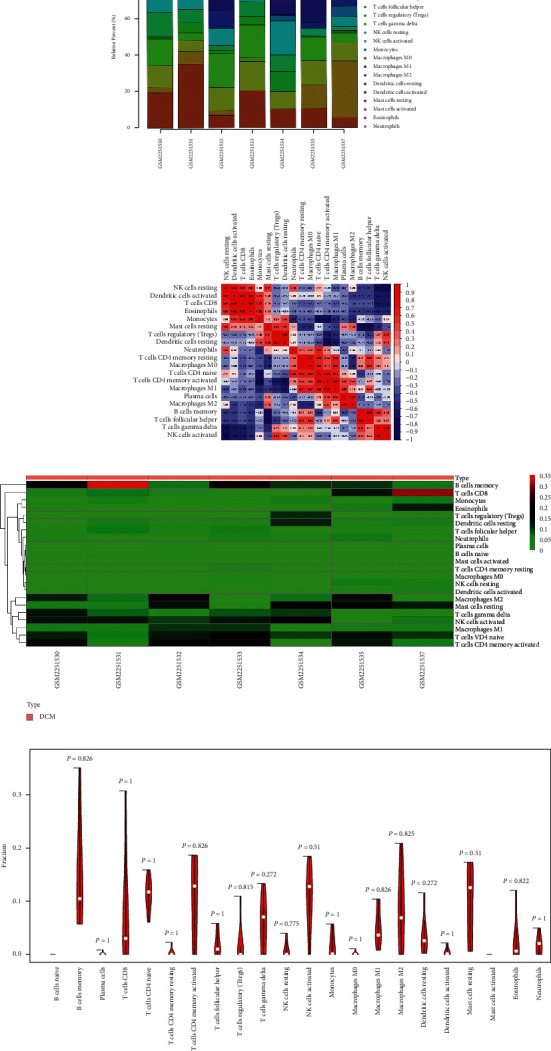
Analysis of immune infiltration pattern in DCM by CIBERSORT.

**Figure 12:**
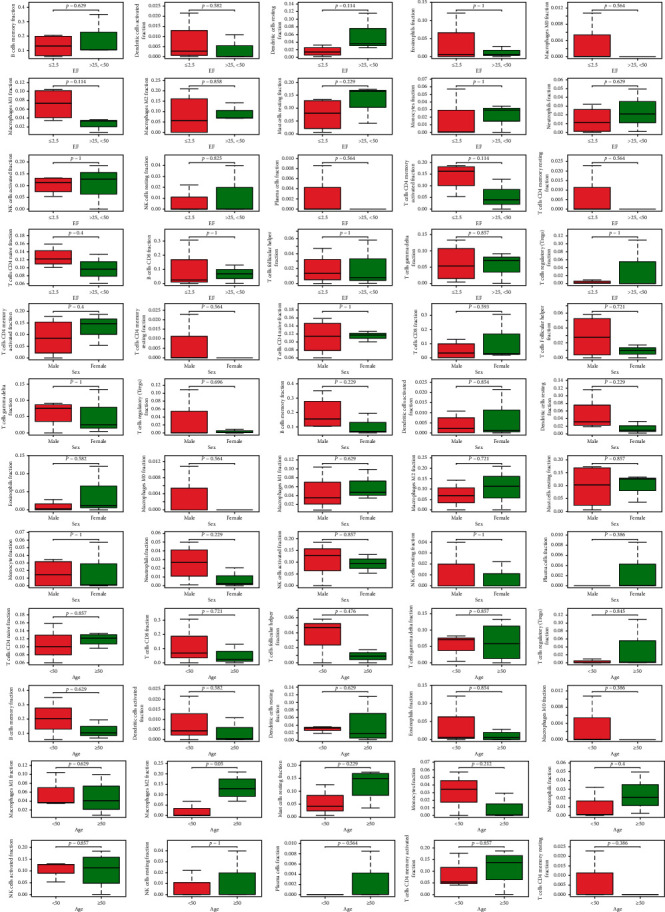
Correlation between each immune cell and clinical characteristics.

Due to technical limitations, the immune infiltration pattern of DCM has not been fully revealed, especially in subpopulations with low cell abundance. Using the CIBERSORT algorithm, we examined differences in immune infiltration in 22 immune cell subsets between DCM and normal myocardium. After filtering according to a *P* value<0.05, immune infiltration of the myocardium in 7 DCM samples was obtained (Figure 11(a)). Some immune cells showed a high normal correlation, such as activated NK cells, dense activated cells, CD8 T cells, eosinophils, and monocytes (Figure 11(b)). The proportion of immune cells in the DCM myocardium exhibited significant group bias clustering and individual differences (Figure 11(c)). Compared to normal tissues, DCM myocardial tissues usually contained a higher proportion of memory B cells, CD8 T cells, activated memory CD4 T cells, activated NK cells, M2 macrophages, and resting mast cells (Figure 11(d)). At the same time, according to the Wilcoxon correlation test, there was no significant correlation between this pattern of immune infiltration and sex, age, or EF value (Figure 12).

### 3.5. PPI Network, GO, KEGG, and Immune-Related Mechanisms of DMCTGs

**Figure 13:**
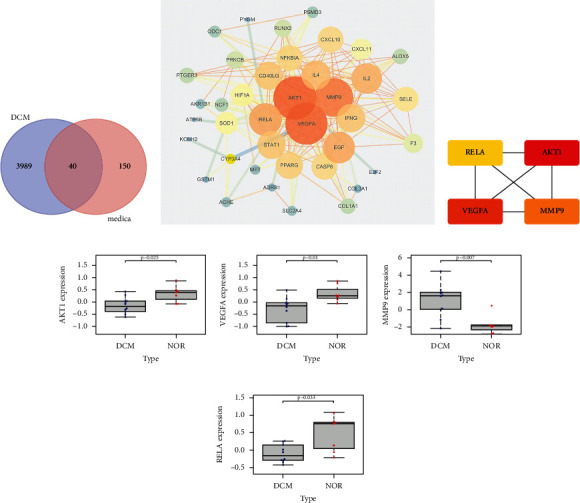
PPI network and hub genes of Astragalus membranaceus in the treatment of DCM.

Forty common target genes of drugs that treat disease (DMCTGs) were obtained from the intersection of the effective component targets of Astragalus membranaceus and DEGs (Figure 13(a)). DMCTGs were imported into the STRING database, and *Homo sapiens* were selected to obtain a PPI network diagram. After using Cytoscape, the node size and line width were positively correlated with the degree value (Figure 13(b)).

**Figure 14:**
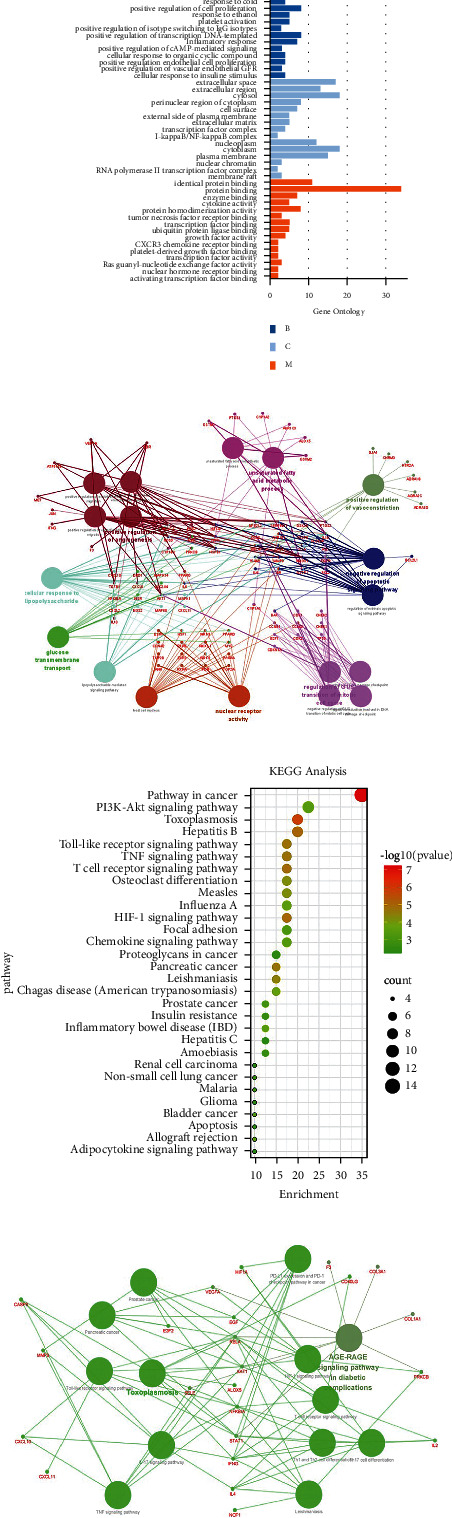
GO/KEGG analysis of DMCTGs.

The top four genes with degree values were AKT1, VEGFA, MMP9, and RELA, with degree values of 16.994, 16.871, 16.616, and 15.774, respectively (Figure 13(c)). The expression levels of these four genes were significantly different between the two groups (*P* < 0.05) (Figures 13(d)–13(g)). The common genes of disease-targeting drugs were introduced into the DAVID database, and “*Homo sapiens*” was selected to obtain GO enrichment, which primarily involved BP, extractive space, negative regulation of the apoptotic process, response to cold, and positive regulation of cell promotion; CC included extracellular region, cytosol, perinuclear region of cytoplasm, and cell surface; and MF included identity protein binding, protein binding, enzyme binding, cytokine activity, and protein homeostasis activity (Figure 14(a)). KEGG enrichment analysis revealed pathways in cancer, toxoplasmosis, hepatitis B, HIF-1 signaling pathway, T cell receptor signaling pathway, toll-like receptor signaling pathway, TNF signaling pathway, and others (Figure 14(c)). The enrichment relationship between specific GO/KEGG entries and genes is shown in Figures 14(b) and 14(d).

**Figure 15:**
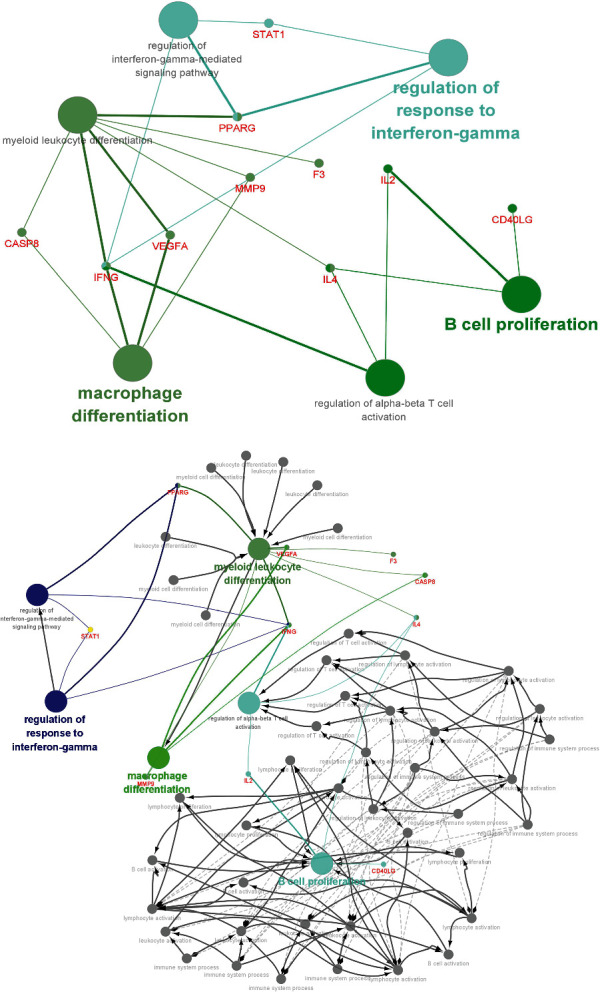
Immune mechanism of Astragalus membranaceus in the treatment of DCM.

The common genes of disease-targeting drugs were introduced into Cytoscape software. The GO/immune system process analysis function was selected using the ClueGO plug-in to obtain the immune-related mechanism of Astragalus intervention in DCM (Figure 15(a)). It primarily involved immune mechanisms, such as myoid leukocyte differentiation, macrophage differentiation, B cell promotion, regulation of alpha-beta T cell activation, regulation of response to interferon-gamma, and regulation of interferon gamma mediated signaling pathways. Each immune cell interacts and come in contact with each other in many indirect or direct ways, forming a complex network of immune regulatory mechanisms (Figure 15(b)). For example, VEGFA regulates myoid leucocyte differentiation and macrophage differentiation simultaneously, and myoid leucocyte differentiation can regulate macrophage differentiation at other levels.

### 3.6. Establishment of a Mechanism Network

**Figure 16:**
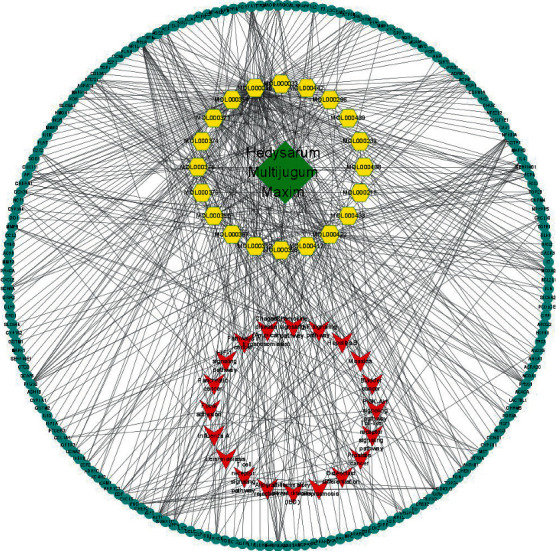
The network mechanism of Astragalus in DCM intervention.

In order to clarify the relationship between the targets and pathways of the cure effects of Astragalus active ingredients treating DCM, Cytoscape software was used to build a path-active ingredient-core targeting network (Figure 16). The network diagram contains both nodes and edges. The green nodes represent the name of the TCM, the yellow nodes represent drug's active ingredient, the blue nodes represent the target gene, and the red nodes represent the signaling pathway. Edges represent the connections between nodes, and more the edges, the more significant the role of nodes in the network graph. The network diagram intuitively shows that Astragalus membranaceus exerts coordinated action on DCM through multiple components, targets, and pathways.

### 3.7. Molecular Docking Verification

**Figure 17:**
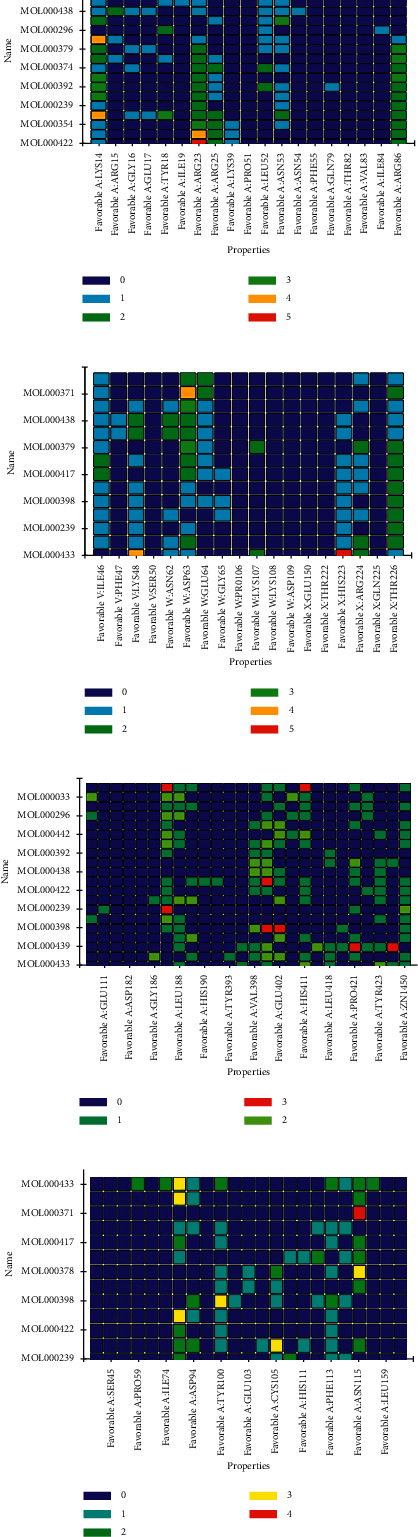
Thermographic analysis of molecular docking.

**Figure 18:**
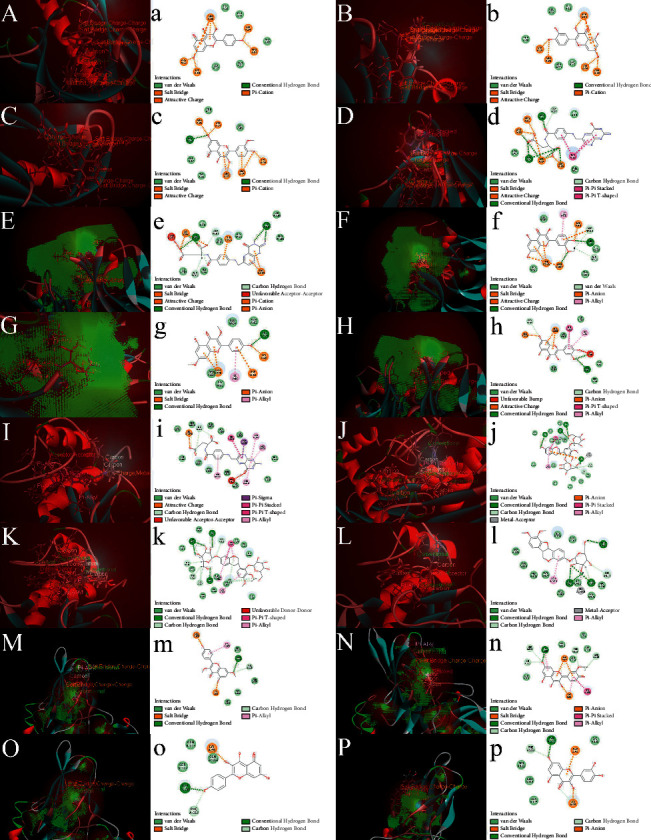
Two-dimensional and three-dimensional pictures of molecular docking conformation: (A–P) top 4 3D docking diagram of AKT1, VEGFA, MMP9, and RELA; (a–p) top 4 2D docking diagram of AKT1, VEGFA, MMP9, and RELA.

The molecular docking verification of the HUB gene in the PPI network of the DCM common gene of Astragalus membranaceus showed that there were 20 AKT1 docking conformations, the top four of which were MOL000422, MOL000098, MOL000354, and MOL000433 with docking scores of 109.459, 108.772, 108.458, and 92.5924, respectively (Figure 17(a)). There were 14 docking conformations of VEGFA, and the top four were MOL000433, MOL000354, MOL000239, and MOL000098 with docking scores of 66.0825, 54.1987, 52.4759, and 51.1214, respectively (Figure 17(b)). There were 20 docking conformations of MMP9, among which the top four were MOL000433, MOL000374, MOL000439, and MOL000379 with docking scores of 83.9275, 71.7529, 67.7594, and 57.9397, respectively (Figure 17(c)). There were 13 docking conformations of RELA, and the top four were MOL000239, MOL000354, MOL000422, and MOL000098, with docking scores of 56.4378, 41.6266, 38.4104, and 37.1788, respectively (Figure 17(d)). A heat map shows the strength of all the binding forces. The vertical axis is the ligand, and the horizontal axis is the protein group. The specific conformation of each group that is satisfactory for docking and fruiting is shown in a three-dimensional and two-dimensional diagram in Figure 18. The three-dimensional conformation primarily shows the spatial position of the ligand in the protein cavity. In contrast, the two-dimensional conformation shows the interaction between the ligand and protein, such as phosphate bonds and hydrogen bonds. At the same time, the name and position of the protein group are also shown in the two-dimensional diagram.

## 4. Discussion

DCM is a heterogeneous type of cardiomyopathy, and the etiology of DCM is divided into primary and secondary groups based on genetics [51]. Primary DCM is mainly related to genetic factors, while secondary DCM refers to systemic disease involving the myocardium, of which cardiomyopathy is only a part of the systemic disease [52]. Currently, the causes of DCM are known to include viral infection, noninfectious inflammatory reactions, endocrine and metabolic disorders, poisoning, autoimmune dysfunction, gene mutation, and genetic factors, which lead to damage to heart morphology and function, resulting in unilateral or bilateral ventricular enlargement, accompanied by ventricular systolic dysfunction, arrhythmia, varying degrees of myocardial hypertrophy, myocardial fibrosis, heart failure, and other symptoms, sudden death or embolism, and other complications [53, 54].

The onset of DCM is relatively insidious, and patients often come to the hospital when they have clinical symptoms, such as arrhythmia or even heart failure. The disease progresses quickly, and the fatality rate is high, which has always been a problem for clinical treatment [55, 56]. At present, modern medicine primarily focuses on symptomatic treatment and the improvement of patients' clinical symptoms. Some treatment methods, such as stem cell therapy and immunotherapy, have emerged in recent years, but they are both expensive and unsatisfactory in clinical treatment at present [57, 58]. TCM has accumulated rich clinical experience in treating DCM based on syndrome differentiation. The etiology, pathogenesis, syndrome differentiation, and treatment of DCM-related diseases have been recorded in ancient Chinese medical literature, and good clinical efficacy has been achieved [59, 60]. At present, TCM treatment of DCM is a new research and development direction with considerable advantages for treating and improving symptoms and the prognosis of patients with DCM [61].

In this study, gene chip data of 17 samples from GSE84796 were obtained from the GEO database, including seven samples of normal cardiac tissue and ten samples of DCM cardiac tissue. Through batch correction and differential analysis, 4029 differential genes were identified, including 1855 upregulated genes and 2174 downregulated genes. GO enrichment analysis showed that the pathogenesis of DCM involved a variety of cellular components inside and outside the cell and nucleus, suggesting that disease pathogenesis results from multiple levels of synergism. Several biological processes are involved in the differentiation, proliferation, maturation, activation, and other processes of immune cells, such as T cells. KEGG pathway enrichment results suggested T cell receptor signaling pathway, Th17 cell differentiation, and intestinal immune network for IgA production immune-related signaling pathway. These results indicated that immune cells, such as T cells, play an essential role in the pathogenesis of DCM, consistent with the conclusion of some previous studies. Jianwu et al. found that in patients with DCM, CD4+ T cells exhibited immune dysfunction and glycolytic metabolic reprogramming based on extracellular acidification and the oxygen consumption rate. Similar results were observed in the spleen and heart CD4+ T cells of autoimmune-induced DCM mice. In vitro, the glycolytic inhibitor 2-deoxy-D-glucose (2-DG) reverses T cell dysfunction. Thus, enhanced metabolic activity directly controls the immune status of CD4+ T cells. The adoptive transfer of CD4+ T cells from DCM mice to normal receptors causes cardiac remodeling and cardiac T cell dysfunction [62]. Inflammation is crucial in the early development and progression of many cardiovascular diseases involving congenital and adaptive immune responses. The role of regulatory T (TREG) cells in inflammation and immune regulation has received increasing attention. The TREG cells play an essential role in inducing and maintaining immune homeostasis and tolerance. The generation or dysfunction of Treg cells can trigger abnormal immune responses and lead to pathology [62]. Myocardial diseases, such as DCM, are characterized by chronic inflammation, mainly mediated by T lymphocytes, and associated enhanced reactive fibrosis [21]. Thus, it is entirely possible to generate chronic autoimmune T cell responses that persist in heart autoantigens through memory T cell responses following a single event leading to cardiac injuries, such as ischemia or infection. These persistent, chronic cardiac aggressive T-cell responses likely predispose patients to physiological decompensation and DCM over time [63].

The idea of gene set enrichment analysis (GSEA) arised from MIT and the Harvard University broad institute research team, which developed genome-wide expression profile chip data analyzing tools. The basic idea is to take a predefined set of genes, rank those genes according to how differentially expressed they are between two types of samples, and then test whether the predefined set of genes is concentrated at the top or bottom of the list. Gene collection enrichment analysis detects gene collection rather than individual gene expression changes, so it includes these subtle expression changes and is expected to achieve more ideal results [35]. In the GSEA of this study, immune-related signaling pathways were more obviously enriched, including the signal regulation pathways of immune cells, such as B cells and T cells, and the action pathways of some immune cytokines, such as antigen processing and presentation, cell adhesion molecules (CAMs), and chemokine signaling pathways. For example, Van der Borght et al. created autoimmune myocarditis experimental mice by adding *α*-myosin-loaded bone marrow antigen-presenting cells into GM-CSF culture and found that myocarditis promoted infiltration of dendritic cells and monocytes in the heart and the self-antigen presentation of conventional type 2 dendritic cells [64].

We also used WGCNA technology to extract modules of the DCM gene, and a total of 4 modules were obtained, among which the blue module was relatively related to the three clinical traits. Next, we performed PPI network analysis on the blue module genes and identified the top 15 genes that formed network associations: ADCY7, Bank1, CD1E, CD19, CD38, CD300LF, Clec4E, FLT3, GPR18, HCAR3, IRF4, LAMP3, MRC1, SYK, and TLR8. Evidence supports the critical role of Bank1 in innate immune signaling of B cells, and the functional difference between the two Bank1 subtypes, the absence of the TIR domain in Bank1-D2, is for its lysine (K) 63-linked polyubiquitination and its ability to produce interleukin (IL)-8 [65]. CD1E, CD19, CD38, and CD300LF proteins synthesize the second messenger of glucose-induced insulin secretion, cyclic ADP-ribose, and nicotinic acid-adenine dinucleotide phosphoric acid. It is also found in thymocytes, proB cells, germinal center B cells, mitogen-activated T cells, IG-secreting plasma cells, monocytes, NK cells, erythrocytes, and myeloid progenitor cells in the bone marrow and brain cells. These proteins are important messenger factors that mediate immune regulation after activation of B-cell-based immune cells [66]. Enrichment analysis indicated that the blue module genes were primarily related to the production of inflammatory factors, cell proliferation, cell apoptosis, and other processes. IL-12 and IL2 synergistically enhance the pathogenicity of myocardial myosin-specific T lymphocytes (MSTLs) [67]. Studies have shown that IL-10-secreting B cells are significantly elevated in the peripheral blood of patients with DCM, suggesting that IL-10-secreting B cells may play an essential role in the pathogenesis of DCM. Based on these 15 genes, we identified three molecular subtypes of DCM [68]. In a clinical meta-analysis of 8097 patients, we found a genotypic and phenotypic association in patients with DCM, including a higher prevalence of sudden cardiac death (SCD), heart transplantation, or ventricular arrhythmias in patients with LMNA and PLN mutations compared to those with segmental gene mutations [10].

However, we found no significant correlation between subtypes and clinical traits in this study. For this reason, the DCM samples considered for inclusion in this study were primarily patients with end-stage DCM. Although the sex, age, and EF value of patients were different, the overall cardiac function and hemodynamic indexes were very unsatisfactory, so we speculate that the immune infiltration and myocardial fibrosis in patients with end-stage DCM are fairly homogeneous. In addition, the initial pathogenesis of DCM samples included in this study was relatively simple, which may be another reason for the single immune infiltration of DCM in the final stage. In a prospective study, DCM was associated with more favorable long-term outcomes in women than men, and gender has become an essential independent predictive factor [69]. Generally, the survival time of children with DCM is usually short, and the patients included in this study were all middle-aged and elderly, so the inclusion condition of this sample reduced the influence of age on differences in immune infiltration patterns to a certain extent [70].

Based on the CIBERSORT algorithm, we systematically analyzed the immune infiltration patterns in DCM and obtained the distribution spectra of 22 immune cell subsets. Compared to the normal myocardial tissue, immune cells in DCM were increased to varying degrees. Among them, memory B cells, CD8 T cells, activated memory CD4 T cells, activated NK cells, M2 macrophages, resting mast cells, and other immune cell subsets exhibited an apparent increasing trend. CD8 T cells and endothelial cells directly stimulate fibrogenesis by activating cardiac fibroblasts and indirectly stimulate fibrogenesis by synthesizing various fibrotic molecules [71]. CD4+ T cells from DCM patients exhibited increased expression levels of CD25 and CD69 and enhanced anti-CD3/28 reactions, indicating that they were in an activated state. In addition, the downregulation of miR-451a promotes the activation and proliferation of CD4+ T cells by targeting the transcription factor Myc in DCM patients and may contribute to the immune pathogenesis of DCM [72].

In a study of 38 cases of idiopathic DCM by immunohistochemical staining, Kanda et al. found that the number of CD57-positive NK cells in patients with DCM was significantly higher than that in the control group. There were functional abnormalities in the NK cell subsets in patients with DCM, and these abnormalities might be related to the pathogenesis of DCM. The quantity of several NK cell subsets (CD16+, CD57+, CD16+CD57+ and CD8+CD57+ cells) positively correlated with the NK cell activity [73]. Despite many excellent studies, the regulatory role of natural killer (NK) cells in the pathogenesis of inflammatory heart disease has been greatly underestimated. Clinical abnormalities in the number and function of NK cells have been observed in myocarditis and inflammatory DCM (DCMI) and heart transplant rejection. Blocking NK cells and their receptors prevent inflammation and destruction in animal models of heart injury and inflammation. In these models, NK cells inhibit the maturation and transport of inflammatory cells, alter the environment of local cytokines and chemokines, and induce apoptosis of nearby resident and hematopoietic cells [74].

Based on the holistic concept, TCM has certain advantages for the treatment of complex diseases. However, TCM's curing mechanism with multiple components, targets, and channels restricts further development and promotion [75]. Current reductionist research strategies still have difficulty uncovering the veil of holistic medicine [76]. Network pharmacology can generate complex interaction networks based on target molecules, biological functions, and bioactive compounds, which is in line with the natural characteristics of TCM and can systematically clarify the mechanism of action of TCM at the molecular level, gradually becoming an overall strategy with a bright future [77, 78]. In addition, in discovering the active ingredients in TCM, web-based methods are expected to break through our understanding of drug action across multiple information layers. Network pharmacology, which considers drug responses in the context of cellular or phenotypic networks, is an alternative to traditional reductionist approaches [79]. This method effectively bridges the gap between modern medicine and TCM and significantly promotes research on the synergistic effects of TCM [80].

The TCMSP database is a TCM information system established by the China Pharmaceutical University. It consists of all 499 Chinese herbal medicines registered in the Chinese Pharmacopoeia, containing 29,384 ingredients, 3,311 targets, and 837 related diseases. In this study, 20 active components of Astragalus membranaceus were obtained from TCMSP, corresponding to 190 targets. Through the PPI network analysis of DMCTGS, the top four genes with degree values were obtained: AKT1, VEGFA, MMP9, and RELA. AKT1 can participate in apoptosis and other processes and plays a vital role in regulating cardiac function and myocardial angiogenesis [81]. VEGFA regulates cardiac microvascular and coronary artery compensation and has a bidirectional regulatory effect on cardiac structural remodeling [82–84]. MMP9 is an upstream regulator of VEGFA [85]. A key source of MMP9 is infiltrating macrophages, and aneurysms form in MMP9 knockout mice after infusion of mouse bone marrow cells with the MMP9 gene [86]. MMP9 is secreted from cells to the extracellular space in the form of a proenzyme. MMP9 becomes active in vitro through the reaction of organomercury preparation, but, in vivo, it requires a series of protease cascades to be activated [87]. MMP9 decomposes a 62-amino acid peptide from interleukin 8 (CXCL8/CL8), increasing its chemotactic activity to neutrophils by a factor of 10. It also inhibits chemotactic factors of other neutrophils [88, 89]. MMP9 binds to CD44 to release stored TGF-*β*1. In addition, MMP9 participates in angiogenesis by releasing the vascular endothelial growth factor (VEGF) [90]. RELA is a crucial regulatory factor of NF-kappaB, which is closely related to cardiovascular disease and involved in various inflammatory responses [91, 92]. The results of DMCTGS enrichment analysis suggested that Astragalus membranaceus exerts various molecular biological functions and participates in a variety of biological processes at multiple cellular component levels. DCM intervention comprises of a multicomponent, multilevel, and multitarget network mechanism. Kaempferol (25 mg/kg) normalized the intestinal antioxidant activity of cold-stressed animals. Kaempferol treatment prevents the cold stress-induced reduction in CD4+T cells in the blood and reduced CD8+T cell levels in mice. In addition, improved hematological characteristics were observed in cows treated with kaempferol [93]. Kaempferol reduces the immune function of dendritic cells and has the potential to treat chronic inflammatory and autoimmune diseases [94]. Kaempferol helps in inhibiting activated proinflammatory cytokines IL-9, IL-13, and CD8+T and neurochemicals and increases anti-inflammatory cytokines and CD4+T levels [95]. Isorhamnetin is the potential molecular basis of Astragalus membranaceus to supplement Qigong. Isorhamnetin is involved in signaling pathways of G-protein coupled receptor proteins, regulation of lipid metabolism, positive regulation of nitrogen compounds metabolism, positive regulation of programmed cell death, fatty acid metabolism, and other biological processes, such as regulating immune function, strengthening the heart, and protecting cardiomyocytes. It improves the pharmacological effects of substance metabolism and antioxidant effects [96]. Isorhamnetin is an effective inhibitor of dendritic cell maturation and transport and significantly reduces TNF-*α*, IL-1*β*, and IL-6 concentrations while inhibiting NF-*κ*B signaling activation [97, 98]. Somasundar Arumugam used a rat model of EAM induced by porcine cardiac myosin and showed that rats subjected to endoreticular structure (ER) stress and adverse cardiac remodeling in the form of myocardial fibrosis after myocarditis were protected from these changes by quercetin treatment [99]. Studies have shown that quercetin improves EAM at least in part by interfering with the production of proinflammatory cytokines (TNF-*α* and IL-17) and anti-inflammatory cytokines (IL-10) [100].

The ClueGo plug-in is a simple and effective gene annotation software designed by Cytoscape. ClueGo can be used for systematic analysis of high-throughput genes and hierarchical ontology tree construction. In this study, the immune mechanism analysis function of Astragalus was used to determine the immune-related mechanism of Astragalus interfering with DCM and the regulatory relationship among the mechanisms. By comparing the immune infiltration pattern of DCM, we believe that the activation of immune cells, such as B cells and T cells, and the secretion of cytokines are potential immune mechanisms by which Astragalus membranaceus interferes with DCM.

Molecular docking technology uses a computer simulation program to predict the possibility and spatial conception of ligand binding to target proteins by defining binding sites [45, 101]. Using molecular docking technology, we can simulate the docking of the effective component and the targets in the component target-signaling pathway network diagram. The analysis of relevant binding parameters and binding conception can be helpful for the discovery of effective components with clinical potential, drug design, and optimization. [102]. In this study, through molecular docking between the active components of Astragalus membranaceus and a HUB protein, various active components of Astragalus membranaceus were obtained that could bind to the active pocket of the HUB protein. Among them, kaempferol, FA, quercetin, and isorhamnetin are potential effective ingredients with therapeutic significance. Molecular docking results also revealed that AKT1, VEGFA, MMP9, and RELA were promising potential targets for DCM treatment, consistent with the above results of PPI network analysis and immune mechanism analysis and verified the gene enrichment results.

## 5. Conclusions

In this study, 4029 DCM differential genes were obtained, including 1855 upregulated genes and 2174 downregulated genes. GO/KEGG/GSEA suggested that the activation of T cells and B cells was the primary cause of DCM. WGCNA was used to obtain blue modules, including ADCY7, BANK1, CD1E, CD19, CD38, CD300LF, CLEC4E, FLT3, GPR18, HCAR3, IRF4, LAMP3, MRC1, SYK, and TLR8, successfully dividing DCM into three molecular subtypes. Based on the CIBERSORT algorithm, the immune infiltration profile of DCM was analyzed. Any immune cell subtypes, including the abovementioned immune cells, exhibited different levels of increased infiltration into the myocardial tissue of DCM patients. However, this infiltration pattern did not correlate with clinical characteristics, such as age, EF, and sex. Based on network pharmacology and ClueGO, 20 active components of Astragalus membranaceus and 40 components of DMCTGS were identified. By analyzing the immune regulatory network, we found that Astragalus membranaceus effectively regulates the activation of immune cells (such as B cells and T cells), cytokine secretion, and other processes and can intervene in DCM at multiple components, targets, and levels. The above mechanisms were verified by molecular docking results, which confirmed that Akt1, VEGFA, MMP9, and RELA were promising potential targets for DCM treatment.

## Figures and Tables

**Table 1:** Effective components of Astragalus membranaceus.

MOL_ID	MOL_NAME	OB	DL
MOL000211	Mairin	55.38	0.78
MOL000239	Jaranol	50.83	0.29
MOL000296	Hederagenin	36.91	0.75
MOL000033	(3S,8S,9S,10R,13R,14S,17R)-10,13-dimethyl-17-[(2R,5S)-5-propan-2-yloctan-2-yl]-2,3,4,7,8,9,11,12,14,15,16,17-dodecahydro-1H-cyclopenta[a]phenanthren-3-ol	36.23	0.78
MOL000354	Isorhamnetin	49.6	0.31
MOL000371	3,9-Di-O-methylnissolin	53.74	0.48
MOL000374	5′-Hydroxyiso-muronulatol-2′,5′-di-O-glucoside	41.72	0.69
MOL000378	7-O-methylisomucronulatol	74.69	0.3
MOL000379	9,10-Dimethoxypterocarpan-3-O-*β*-D-glucoside	36.74	0.92
MOL000380	(6aR,11aR)-9,10-dimethoxy-6a,11a-dihydro-6H-benzofurano [3,2-c]chromen-3-ol	64.26	0.42
MOL000387	Bifendate	31.1	0.67
MOL000392	Formononetin	69.67	0.21
MOL000398	Isoflavanone	109.99	0.3
MOL000417	Calycosin	47.75	0.24
MOL000422	Kaempferol	41.88	0.24
MOL000433	FA	68.96	0.71
MOL000438	(3R)-3-(2-hydroxy-3,4-dimethoxyphenyl)chroman-7-ol	67.67	0.26
MOL000439	Isomucronulatol-7,2′-di-O-glucosiole	49.28	0.62
MOL000442	1,7-Dihydroxy-3,9-dimethoxy pterocarpene	39.05	0.48
MOL000098	Quercetin	46.43	0.28

## Data Availability

All the data generated or analyzed during this study are included in this article. The materials described in the manuscript, including all relevant raw data, will be freely available to any scientist wishing to use them for noncommercial purposes without breaching participant confidentiality.
